# Oxford Nanopore MinION Sequencing and Genome Assembly

**DOI:** 10.1016/j.gpb.2016.05.004

**Published:** 2016-09-17

**Authors:** Hengyun Lu, Francesca Giordano, Zemin Ning

**Affiliations:** 1 National Centre of Gene Research, Chinese Academy of Sciences, Shanghai 200233, China; 2 The Wellcome Trust Sanger Institute, Wellcome Genome Campus, Hinxton, Cambridge CB10 1SA, UK

**Keywords:** Third-generation sequencing, Oxford nanopore MinION device, *De novo* assembly, Structural variations, Molecular clinical diagnostics

## Abstract

The revolution of genome sequencing is continuing after the successful second-generation sequencing (SGS) technology. The **third-generation sequencing** (TGS) technology, led by Pacific Biosciences (PacBio), is progressing rapidly, moving from a technology once only capable of providing data for small genome analysis, or for performing targeted screening, to one that promises high quality ***de novo* assembly** and **structural variation** detection for human-sized genomes. In 2014, the MinION, the first commercial sequencer using nanopore technology, was released by Oxford Nanopore Technologies (ONT). MinION identifies DNA bases by measuring the changes in electrical conductivity generated as DNA strands pass through a biological pore. Its portability, affordability, and speed in data production makes it suitable for real-time applications, the release of the long read sequencer MinION has thus generated much excitement and interest in the genomics community. While *de novo* genome assemblies can be cheaply produced from SGS data, assembly continuity is often relatively poor, due to the limited ability of short reads to handle long repeats. Assembly quality can be greatly improved by using TGS long reads, since repetitive regions can be easily expanded into using longer sequencing lengths, despite having higher error rates at the base level. The potential of nanopore sequencing has been demonstrated by various studies in genome surveillance at locations where rapid and reliable sequencing is needed, but where resources are limited.

## Introduction

Genomics has been revolutionized over the last 20 years by the development of first- and second-generation sequencing (SGS) technologies, enabling the completion of, among many other notable projects, the Human Genome Project [1], [2] and the 1000 Genomes Project [3]. The first method to sequence DNA was developed by Sanger in 1975 [4], [5], and another method was developed in parallel by Maxam and Gilbert in 1977 [6], which were termed the “chain-termination method” and the “chemical sequencing method”, respectively. Being less complex and more scalable in comparison to the chemical sequencing method, the chain-termination method eventually was used extensively for first-generation sequencing. DNA libraries with different insert sizes can be easily generated, which turns out to be useful as whole genome assembly benefits from the reads with mixed insert sizes. However, the low throughput and high cost of the first-generation sequencing led to a fundamental shift in methodology, taking us to SGS. The revolution of massively parallel sequencing started in 2005 with the introduction of Roche 454’s pyrosequencing system. It was then followed by the sequencing-by-synthesis based Genome Analyzer platform from Illumina/Solexa in 2007, and by ABI’s SOLiD system using sequencing-by-ligation [7]. SGS technologies have dominated the sequencing market in the last decade, owing to its ability to produce an enormous volume of data cheaply. However, short reads produced by SGS lead to highly-fragmented assemblies when it comes to *de novo* assembly of larger genomes, due to difficulties in resolving repetitive sequences in the genome [7].

The demand for technologies that can operate at higher speed and produce longer reads has resulted in the advent of new sequencing approaches—the so-called third-generation sequencing (TGS). Major SGS platforms adapt sequencing by synthesis (SBS) technologies that rely on PCR to enlarge clusters of a given DNA template. TGS technologies, by contrast, directly target single DNA molecules, enabling real-time sequencing, where reads are available for analysis as soon as they have passed through the sequencer. There are three important improvements in TGS platforms: (1) increase in read length from tens of bases to tens of thousands of bases per read; (2) reduction of sequencing time from days to hours (or to minutes for real-time applications); and (3) reduction or elimination of sequencing biases introduced by PCR amplification [8]. Apart from Helicos fluorescent sequencing (http://seqll.com/), the first successful single-molecule real time (SMRT) technology was introduced by Pacific Biosciences (PacBio, http://www.pacb.com/). With modern reagents and sequencing kits, the typical throughput of the PacBio RS II system is 0.5–1 gigabytes (GB) per SMRT cell, with a mean read length of roughly 10 kilobases (kb). Nonetheless, PacBio reads have a significantly higher error rate (∼10%−15%) than SGS reads (<2%) [9]. Fortunately these sequencing errors are randomly distributed, the rates can therefore be greatly reduced through the use of circular consensus sequencing (CCS) [10], where a single molecule template and its complement strand are sequenced multiple times to generate a unique consensus.

In 2014 Oxford Nanopore Technologies (ONT) released a new TGS platform, the MinION device, through an early access program (The MinION Access Program, MAP). The read length profile of the ONT data is very similar to that of PacBio, with a maximum length up to a few hundred thousand base pairs [11], [12]. However, ONT reads have error rates higher than PacBio reads, with accuracy ranging 65%−88% [11], [12], [13]. In addition, the throughput per MinION flowcell run is not very stable at the moment, varying from below 0.1 GB to 1 GB of raw sequence data [13]. Due to its small size and low equipment cost, the MinION sequencer is attracting considerable interest in the genomics community, particularly for pathogen surveillance and clinical diagnostic applications, as these areas would benefit from the real time nature of this sequencing platform. A comprehensive review of PacBio sequencing and its applications has been presented by Rhoads and Au [14], who also compared the performance of PacBio sequencing with SGS platforms. In this review, we focus on MinION sequencing, the data characteristics, the algorithms for genome assembly, and the differences between the PacBio and ONT platforms.

## The MinION device

**Figure 1:**
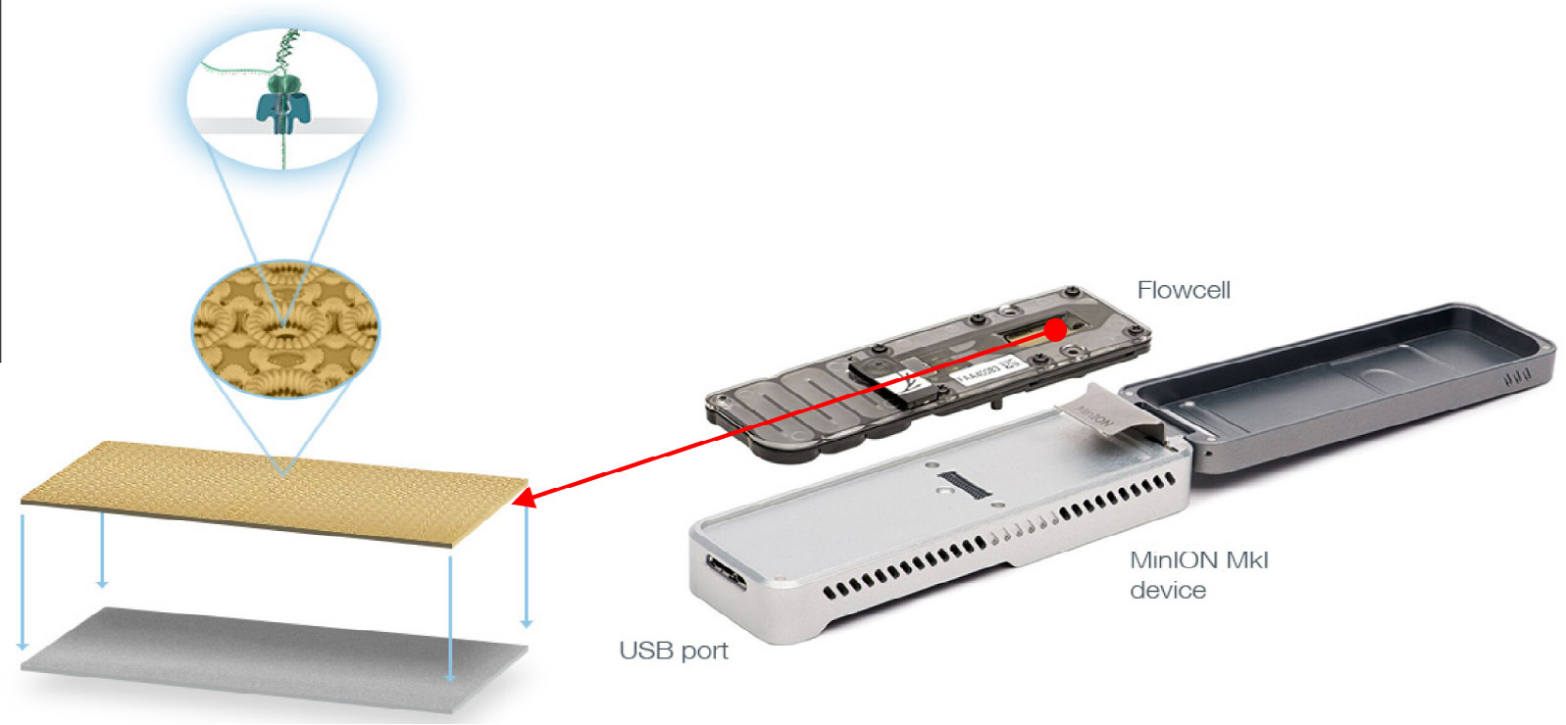
**The MinION sequencing device** DNA sequencing is performed by adding the sample to the flowcell. When DNA molecules pass through or near the nanopore, there will be a change in the magnitude of the current in the nanopore, which is measured by a sensor. The data streams are passed to the ASIC and MinKNOW, the software that generates the signal-level data. ASIC, application-specific integrated circuit.

Measuring only 10 × 3 × 2 cm and weighing just 90 g, the MinION is the smallest sequencing device currently available (Figure 1). It can plug directly into a standard USB3 port on a computer with low hardware requirement and simple configuration. Using Window 7 or 8, a computer with a solid-state drive (SSD), more than 8 GB of RAM and more than 128 GB of hard disk space (though 1 terabytes is recommended) can be used for sequencing. Specialist software called MinKNOW runs on the host computer to which the MinION is connected. MinKNOW carries out several core tasks—data acquisition; real-time analysis and feedback; data streaming while providing device control (including run parameter selection); as well as sample identification and tracking—thus ensuring that the platform chemistry performs correctly in processing the samples. Only high-quality genomic DNA is recommended for the Genomic DNA Sequencing Kits (at the time of starting this paper, there were only two kits available: SQK-MAP005 and the more recent SQK-MAP006). The starting DNA material should be composed of long fragments (>30 kb), which can be typically obtained using standard extraction and clean-up methods.

**Figure 2:**
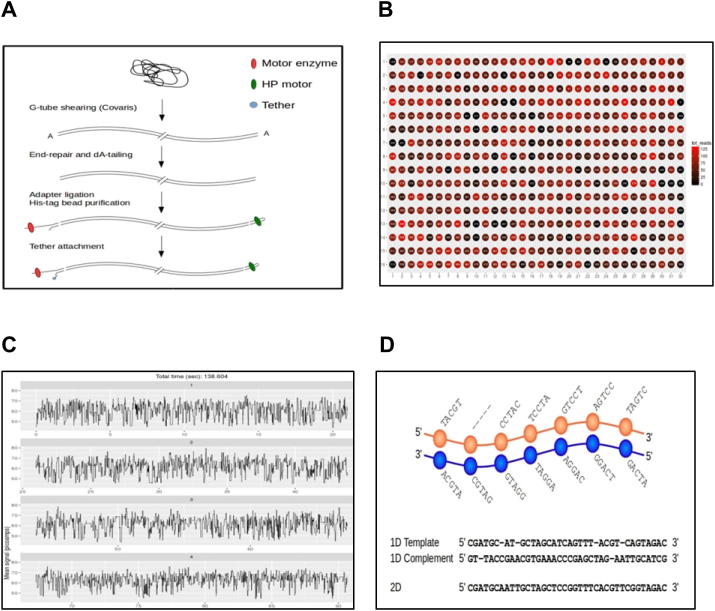
**Flow of Oxford Nanopore sequencing process** **A.** Library preparation schematic for the genomic DNA sequencing kit (SQK-MAP-003). **B.** 512 channels with different levels of activity in a flowcell are shown in different color (most active channels are in red). **C.** “Squiggle plots” of fluctuating electrical signals, which can be translated into DNA bases. **D.** 5-mers decoding from event information and alignment of 1D and 2D base calls.

Similar to SGS technology, library preparation is needed for various applications using MinION. Here, long and double-stranded DNA (dsDNA) should be used so that sequencing of both strands can be performed. The current library construction process consists of a number of steps, which are carried out in the following order: genomic DNA shearing using a Covaris g-TUBE; an optional “PreCR” step to repair damaged DNA; end-repair to create blunt ends in sheared DNA and PCR fragments; dA-tailing to add an “A” base to the 3′ end of the fragment; adapter ligation; and finally His-bead purification to remove nucleotides and enzymes. The library usually contains two adapters, the leader adapter and the hairpin adapter, each ligated to one end of the dsDNA (Figure2**A**). The leader adapter is denoted as the “Y adapter” as it has a “Y” shaped structure, whereas the adapter with hairpin structure is called the “HP adapter”. Sequencing begins at the single-stranded 5′ end of the Y adapter, followed by the “template” strand, then the HP adapter, and the “complementary” strand. The motor protein starts to unzip the dsDNA when approaching the turning point of the Y adapter complementary region. At this point, the first (the “template”) strand is passed into the nanopore with speed determined by the motor protein. Once the HP adapter is reached, a protein (the “hairpin protein”) allows the complementary strand of the DNA to be passed through the nanopore in a similar manner. Base-calling can then be performed. If information from only one strand is used, the base-calling is termed 1-directional (1D); whereas a 2D base-calling is performed, if information from both strands is incorporated, which results in higher base quality.

On the membrane with nanopores incorporated, a voltage can be applied to drive DNA through the pore and an ion current flow can be measured. When a DNA molecule passes through the nanopore, a change of the current in pattern or magnitude can be observed and characterized. The current in the nanopore is measured by a sensor several thousand times per second, and the data streams are passed to a microchip called the application-specific integrated circuit (ASIC). Finally, data processing is carried out by the MinKNOW software, which deals with data acquisition and analysis.

The current MinION flowcell has 512 channels, allowing up to 512 independent DNA molecules to be sequenced simultaneously [13]. Each channel is connected to four wells and can provide data from one of the four wells at a time. The performance in terms of data production (*i*.*e*., the number of reads produced) varies from channel to channel, since some pores are more active than others. Data from a typical run are shown in Figure2**B**. Channels in red produced up to 126 reads, whereas some channels (those shown in black) produced zero reads. In order to reduce noise, the raw current measurements are processed and converted into a sequence of ‘events’, each with a mean value for the current and an associated variance and duration. The so-called “squiggle plot” shown in Figure2**C** shows the raw current measurements over time. As DNA passing the pore can contribute to the change in the ion current, base-calling is performed on 5-mers or 6-mers. There are 4^5^ = 1024 possible combinations and 4^6^ = 4096 when 5-mers or 6-mers are used, respectively. An optimal path, shown in Figure2**D**, is searched through a hidden Markov model (HMM) of successive 5-mers (or 6-mers) using the Viterbi algorithm [15].

**Figure 3:**
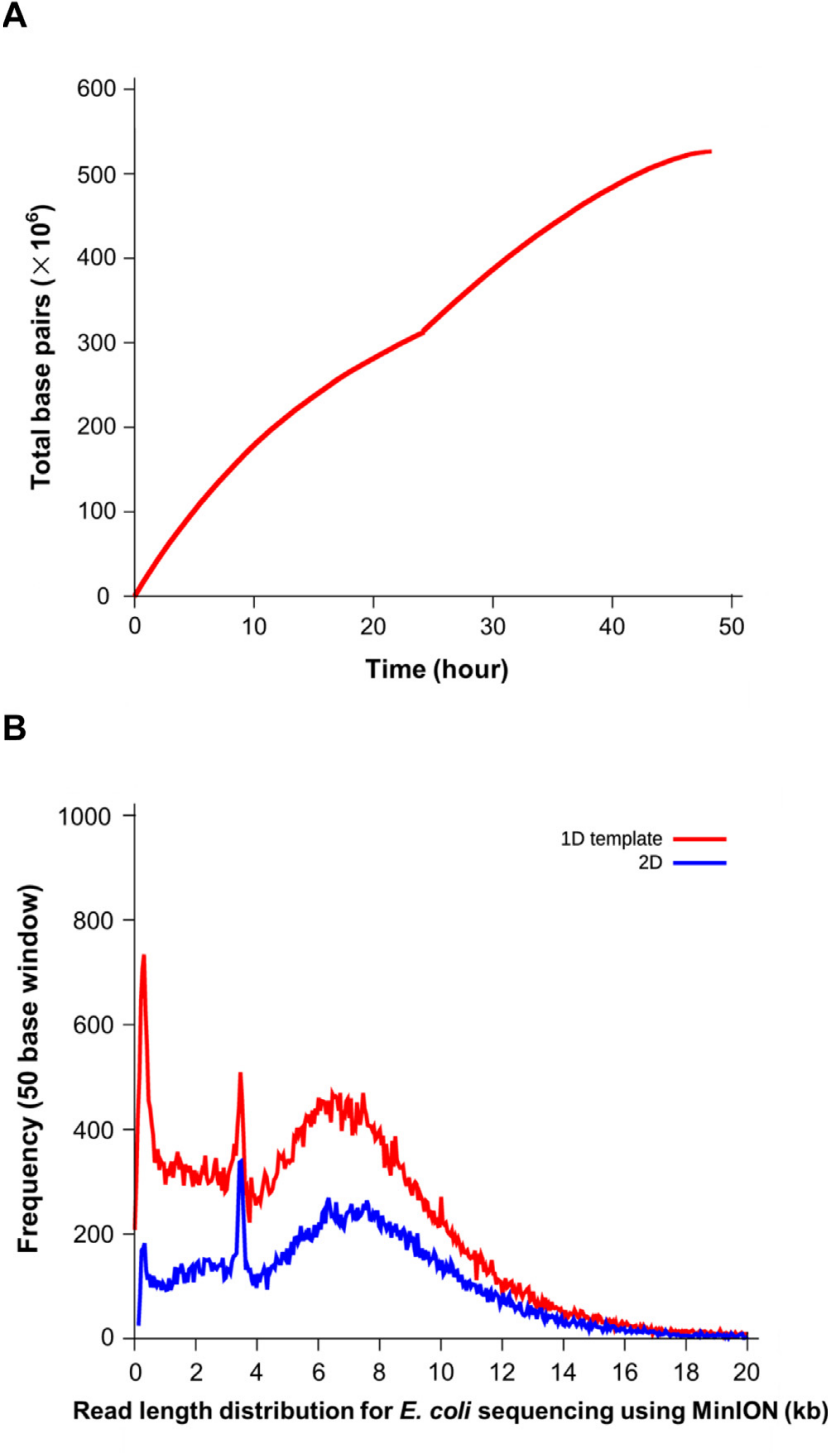
**Time course of base throughput and read length distribution** **A.** The throughput of bases with time generated using Poretools [23]. **B.** Distribution of read length using data from one flowcell run [13].

Currently, MinION flowcells have 2048 wells—4 for each of the 512 channels. At the beginning of a run, the 4 wells in each channel are tested using a procedure called “Mux”. During this process, each of the four wells in a channel is ranked in terms of activity, with the most active one being placed in a group ‘g1’, the second most active placed in a group ‘g2’, and so on. At the start of a 48-h run (the default run time), each active channel starts taking data from its ‘g1’ well and continues to do so for the first 24 h. The remaining 24 h are then equally distributed between the other 3 groups: at the end of each period, each channel switches to the well contained in the next group. The transition between g1 and g2 pores is clearly visible in Figure3**A**, where the throughput suddenly increases 24 h after the start of the run. The read length profile is shown in Figure3**B**. The protocol for the default 48-h MinION run described here is defined in a combination of Python scripts that are launched at the start of a run. While the original protocol ensures a good performance, some members of the MinION Access Program (MAP) community have already started to test new settings and procedures by customizing these scripts. An example of this customization aimed at increasing a flowcell throughput has been made publicly available for the MAP community by John Tyson (Modified MinKNOW scripts, personal communications).

## Data formats and base-calling

For data analysis, most bioinformatics tools take FASTA or FASTQ files as input, where base-calling has already been done during the sequencing process or off line with the sequencer. For new platforms in their early stages, however, original raw data files may be useful for some applications. Currently, the MinION outputs one FAST5 file per read [13]. Much like the h5 file format adopted by PacBio, the FAST5 file format is based on the hierarchical data format 5 (HDF5) standard (https://www.hdfgroup.org). FAST5 files have a hierarchical structure, meaning that they can store both the metadata associated with a read, along with the events (such as aggregated bulk current measurements) pre-processed by the sequencing device. Each read is produced by one of the MinION’s 512 channels, and the metadata associated with each read are stored in a unique FAST5 file. To ensure the unique identity of each read name, a combination of information such as experiment name and batch, channel, as well as file numbers is used.

Currently, base-calling is carried out in the Amazon cloud, rather than on the local computer linked to the sequencing device. The cloud-based Metrichor [16] is used with the data from each run. This service analyzes the FAST5 files generated by the sequencing software after uploading and returns with the analysis results attached. The current workflow comprises of a number of steps. First, the registered current values for a molecule are analyzed, in order to determine which levels of current correspond to the template strand and which correspond to the complementary strand. This stage is also needed to remove the current values related to the adapters. Next, statistical models trained by ONT are used to establish the relationship between 5-mers and current levels. Differences between the model and the observed current levels are then computed. Finally, two 1D sequences may be obtained for each molecule, from which one 2D sequence is constructed if possible. After base-calling has been carried out, Metrichor classifies the reads into two ‘read classes’: ‘pass’ and ‘fail’. The data in the raw FAST5 file and the metadata and base-calls returned by Metrichor are combined into a base-called FAST5 file, which can be downloaded to a directory chosen by the user.

The procedure of base-calling can be briefly described as follows. (i) Template and complement events are processed separately and then used for 1D base-calling. (ii) If the ratio of template sequence length to complement sequence length after base-calling is between 0.5 and 2.0, 2D base-calling is attempted. (iii) If the 2D base-calling is successful a 2D read is produced. (iv) A quality score (Q-score; developed by ONT) is computed in order to quantify the 2D read quality. The FAST5 is put into a “pass” directory, if the 2D read has a mean Q-score > 9; whereas all other FAST5 files are put into a “fail” directory. Therefore, the “fail” directory includes FAST5 files that have: (a) 2D reads with mean Q-scores ⩽ 9 (in addition to template and complement reads); (b) 1D template and complement reads for which 2D base-calling was not achieved [either the ratio is too big (>2.0)/too small (<0.5) or 2D base-calling failed]; and (c) only a template read.

The reads classified as ‘pass’ can normally be considered the high-quality reads from the experiment. At the time of writing, there are two sequencing kits: SQK-MAP005 (using 5-mers) and SQK-MAP006 (using 6-mers). The first notable difference between these two kits is the sequencing speed, which is doubled from ∼30 bp/s for SQK-MAP005 to ∼75 bp/s for SQK-MAP006, likely resulting in increased yields. However, its effect on base quality remains to be quantified, as an increase in speed may also increase the chance of missing events (transitions between signal levels). As a result, it could potentially increase the number of base deletions after base-calling. The second difference between these two methods is that the number of possible kmer combinations during base-calling has been increased from 4^5^ to 4^6^, meaning that the expected ionic current in each event is estimated to account for the influence of 6 neighboring bases instead of only 5 bases. Therefore, complications in quality profiles could be introduced due to these changes. Although the overall impact on data quality remains to be justified, earlier results from Nick Loman (http://lab.loman.net/2015/09/24/first-sqk-map-006-experiment/) suggest that the accuracy of the assembly is much better with SQK-MAP006 data. Most of this improvement is due to better representation of homopolymer sequences after base event polishing using Nanopolish [17]. It should be noted that the kit SQK-MAP006 is relatively new, and thus all data presented in this review article are generated using SQK-MAP005.

ONT has released several versions of flowcells, where data quality and throughput vary significantly [18]. The release of version R7+ flowcells to the MAP community received positive feedback from different users who were using them for different applications. Norris et al. [18] compared the data quality of different flowcell versions. Using R7 flowcells, the average base accuracy was only 67.4% for the reads produced, with 24.2% mismatched bases, 7.5% insertions, and 8.3% deletions. However, after upgrading to the newer version R7.3, the average base accuracy was increased to 86%, with 9.7% mismatches, 4.2% insertions, and 4.4% deletions, demonstrating a dramatic improvement. It should be noted that the accuracy metrics are computed from alignments of the base calls to the reference, and different alignment methods sometimes produce slightly different estimates. Popular aligners for ONT reads include BWA-MEM [19] (parameters ‘-× ont2d’) and LAST [20] (parameters ‘-s 2 -T 0 -Q 0 -a 1’), as suggested by Nick Loman (personal communication, 2015).

With the improved utility and quality of the MinION data, performance reproducibility of flowcells has been assessed by the MinION Analysis and Reference Consortium (MARC). This investigation is necessary because the variation in how much success individual users have with the MinION and the wide range of usage of the device make it difficult to determine the cause of this variation from individual sequencing runs. In the paper presented by the MARC (also known as MAP) [13], a control strain of *Escherichia coli* K-12 was used by five laboratories from different countries to generate data. Each participating laboratory used QIAGEN Genomic-tip 20/G to extract DNA from approximately 4 × 10^9^ log-phase cells. The day after extraction, a library was prepared using the Genomic DNA Sequencing Kit SQK–MAP005, following the protocol issued by ONT. Sequencing was carried out at each participating laboratory. Despite slight variations in performance being observed, in general there was consistency with regard to base error rate, throughput, and read length [13].

For a rapidly-developing new sequencing technology, collective efforts with community involvement are important. An initial lack of tools for the analysis of data led to the development of a series of bioinformatics packages for exploring the native FAST5 data produced by the Metrichor real time analysis platform. Poretools [21] and poRe [22] were developed to convert and visualize the raw data, NanoOK [23] was developed to assess quality, yield, and accuracy of the data, whereas minoTour [24] can monitor reads produced by the sequencing process in real time. Table 1 lists a number of tools [17] for dedicated nanopore data analysis with various applications. Genome scaffolding tools such as SSPACE-LongRead [32] and SMIS [33] are also included in the table as they are not complete pipelines for *de novo* assemblies. Full details of assembly pipelines will be discussed in the next section.

## *De novo* genome assembly

One of the technical advantages of TGS data is the read length, which offers great prospects for genome assembly. Generally, assemblers are based on several different types of algorithms, such as greedy, overlap-layout-consensus (OLC), de Bruijn graph (DBG), and string graph (reviewed by Henson et al. [34]). While early assemblers usually used the OLC paradigm for assembling Sanger sequencing reads, modern assemblers use DBG paradigm for assembling SGS short reads. Although the DBG approach is faster, OLC-based algorithms perform better for longer reads with higher base error rates [35]. Therefore, assemblers designed for long PacBio and ONT reads are mainly pipelines based on OLC approaches.

To make a *de novo* OLC-based assembly, there are normally three basic stages in the process: preassembly, consensus buildup, and consensus polishing. The goal of preassembly data processing is to generate long and accurate sequences by correcting base errors. Seed reads (a subset of the sequencing reads) are selected based on the read length distribution. Each single read is then mapped to the seed reads to generate a consensus sequence for the mapped reads, resulting in long and accurate fragments of the target genome. The computation in this step is very intensive as it involves all-vs-all raw read mapping and base error correction. The next step is the consensus building from the overlapping reads. There are options available when selecting assembly algorithms, but OLC assemblers offer clear advantages for *de novo* assembly using multi-kb long reads. For genomes with repeats of any length, a single long error-corrected read could simply bridge the gaps of unique sequences and ensure that the consensus building process continues without interruption. When designing a *de novo* genome sequencing project, reasonable read coverage (50–60×) is needed to generate sufficient coverage of reads that uniquely anchor the longest repeat regions in the genome assembly. For preassembled reads, there could be base errors in the repetitive regions, where raw base errors are coupled with repeats. Errors such as indels and substitutions in the preassembled reads could also be easily passed on to the consensus. Therefore, there is a need for consensus polishing for assemblies produced from TGS data. To significantly reduce the remaining number of errors in the draft assembly, a quality-aware consensus algorithm could be implemented to use the rich quality scores embedded in PacBio bas.h5 files or the event-by-event raw ion currents in ONT MinION FAST5 files. The Quiver [36] algorithm processes four different per-base quality values (QV scores) available in the PacBio bas.h5 files, which represent the intrinsically calculated error probabilities for inserted, deleted, substituted and merged base calls in single pass reads. For the MinION platform, the final polishing of the assembly can be performed using Nanopolish, developed by Loman et al. [17], which improves the assembly base quality by re-evaluating and maximizing the probabilities for each base according to the raw ion currents of the events, accessible in the FAST5 files.

## Loman, Quick, and Simpson pipeline

With a similar level of base errors in sequencing reads, assembly pipelines for MinION data use the same strategies as those successfully used for PacBio single molecule long reads. The Loman, Quick and Simpson (LQS) pipeline [17] presents an excellent example on how a high quality assembly could be produced from nanopore sequencing data by following the steps of preassembly data processing, overlap detection, consensus generation, and finally contig base polishing using the event data. The FASTA sequences for reads are extracted using Poretools [21] and converted to the format expected by alignment tool DALIGNER [37]. Potential overlaps between the reads are detected from the DALIGNER alignments. Multiple alignments are computed using the partial-order alignment (POA) software [38], where data inputs are single reads and their overlaps. The use of partial-order graphs leads to consensus sequences with better quality in the presence of indels. To achieve better results, the base error correction process needs to be run multiple times, using the corrected reads as the new input. It has been reported that error correction increased the mean identity percentage from 80.5% to 95.9% after the first iteration, and again to 97.7% on the second iteration [17]. After two rounds of base-error correction, the reads can be assembled using v8.2 of the Celera Assembler [39] from which a draft assembly can be obtained. One of the most interesting features in the LQS pipeline is its stand-alone tool Nanopolish, which polishes the contig sequences in the draft assembly using the ion current information registered by the MinION device. The polishing procedure starts with the initial draft assembly and modifies each 5- or 6-mer by introducing substitutions, insertions, deletions, and substrings sampled from the 2D base-called reads. A probabilistic model of the nanopore sequencing process is then used to evaluate whether the modifications to the kmer words increase the probability of the electric signal data observed during the collection of MinION reads. The aforementioned process of modification and evaluation needs to run iteratively until no more improvements could be seen in the assembly. In the tested example, the *E. coli* K-12 MG1655 chromosome was assembled into a single 4.6-megabase contig. The assembly reconstructs correct gene order and shows 99.5% nucleotide identity after consensus polishing using a probabilistic model of the signal-level data.

## PacBio Corrected Reads assembler and Canu

The PacBio Corrected Reads (PBcR) assembler (http://wgs-assembler.sourceforge.net/wiki/index.php/PBcR) is the first pipeline using hierarchical assembly method (correct, overlap, assemble), which demonstrates that noisy long reads can be used for assembly after base error correction. The hierarchical genome-assembly process (HGAP) [40] was developed for PacBio long reads with inputs of PacBio-specific h5 files [41], which are different from the signal-level data generated by the MinION device. However, when raw sequencing data are converted into FASTQ/FASTA files, alignment tools such as the basic local alignment with successive refinement (BLASR) [42], DALIGNER (https://github.com/thegenemyers/DALIGNER) or Celera Assembler could be used to compute multiple alignments for base error correction and overlap detection followed by consensus layout. Fast and sensitive mapping of error-prone long sequencing reads is crucial and computational demand on multiple alignments is high. Over the past few years, PBcR has gained significant performance improvements in assembling bacterial genomes [43], [44], middle-sized *Drosophila melanogaster* and *Arabidopsis thaliana* genomes [45], and more recently human genomes [46], [47], thanks to the implementation of a new, faster algorithm called MinHash alignment process (MHAP) [45]. MHAP employs a probabilistic approach to overlap-based assembly of long reads. To speed up the assembly of gigabase-sized genomes, the MinHash represents long and sparse text as seeded sequences or a string of information as a set of fingerprints, in such a way that the assembly process is carried out with more compact data using fewer computational resources. Latest efforts have led to the development of a new assembler Canu (https://github.com/marbl/canu), which follows the same strategy and method of those used in PBcR, but offers higher speed and better usability. The first version of Canu was released at the end of 2015.

## Falcon assembler

The Falcon [48] assembler developed by Jason Chin from PacBio is another pipeline adopting the strategy of HGAP. It shares many features with PBcR, such as raw reads overlapping for base error correction using DALIGNER and overlap filtering. The major difference lies in its contig consensus generation. Given the overlapping data, a string graph is constructed from the dataset to represent the connections of those reads with overlaps, where a path is a connection between the reads and an edge is a connected read. Due to genome polymorphism and sequencing errors, graph edge reduction is carried out to remove complexity. The consensus step to create draft contigs is to find a single path corresponding to each contig graph and then to generate sequences accordingly. There are continued efforts to make Falcon a polyploidy-aware assembler and new algorithms have been designed to reconstruct comprehensive haplotypes. For simple graphs where the incoming and outgoing edges are unique, a contig is generated in a straightforward way. In the case that a contig graph is not a simple path, a primary contig is generated first with the end-to-end path that has the most overlapped bases. Furthermore, in a multiple path graph, if an alternative path is different from the primary one is possible, another contig is constructed, which is called an associated contig. Identity comparison between primary and associated contigs can reveal genome polymorphism. When associated contigs are induced by sequencing error, there will be a high identity between the alternative contig and the primary contig (>99% most of time). In the case where there are true structural polymorphisms, primary contigs and associated contigs are separated, each representing a different haplotype from the genome.

## Miniasm assembler

Read error correction is the most CPU-intensive stage of the whole assembly process, and assemblies on gigabase-sized genomes are still out of reach for many projects due to high sequencing costs and large computational requirements. The Miniasm [49] assembler developed by Heng Li takes a different approach to deal with noisy long reads by skipping the step of read error correction completely. The alignments are performed based on all-vs-all read mapping and the resulting alignment file is saved for further processing of assembly graphs. For each read, the first step is to obtain an approximate estimate of read coverage, which is achieved by finding the longest contiguous region covered by three good mappings. The second stage is to use the coverage information to find the good regions again but with more stringent thresholds. A string graph is then computed to store mapped reads, remove weak overlaps, and collapse short bubbles. With a pileup of overlapped reads, the algorithm simply searches for the best path and then concatenates pieces of reads to generate the final unitig sequences. Without base error correction, the per-base error rate of the contigs is similar to that of the raw input reads. Miniasm is the fastest assembler examined in this paper, taking only a few minutes for a bacterial-sized genome. For a dataset of *Caenorhabditis elegans* sequenced at 45× coverage, it only takes 9 min for Miniasm to assemble the genome using 16 CPU cores, achieving an N50 size of 2.8 megabases. Efficient memory usage and high speed in assembly are due to the use of its own hash table-based aligner, minimap [50]. Algorithmically, minimap combines the reduced representations of MHAP using sparse fingerprints and the kmer sorting and sorted lists merging of DALIGNER. Working directly on noisy uncorrected reads, one major concern of Miniasm is how to distinguish the sources which lead to low-identity matches between two noisy reads: is the low identity caused by the stochastically-higher base error rate on reads, or because reads come from two recent segmental duplications? At high read coverage, assembly pipelines using base error correction such as LQS, PBcR, Canu, and Falcon sometimes offer an advantage in separating high error rate from duplications/repeats.

## Hybrid assemblers

The NGS platforms such as Illumina’s HiSeq and MiSeq have played a dominant role in genomic research and applications. It is foreseeable that short read data will continue to be a very important part of data sources for years to come. Different algorithms have been explored for genome assembly and many pipelines have been developed for various applications [7], [34]. With the emergence of TGS, some assemblers start to include long reads into the pipeline to improve assemblies primarily generated from short reads. Typical examples of hybrid assemblers are ALLPATHS-LG [51], Celera Assembler’s PacBio corrected reads pipeline (pacBioToCA [52]), SPAdes [53], and SSPACE-LongRead [32]. Assessment and evaluation on these hybrid methods have been reported by Utturkar et al. [54], Liao et al. [55], and Sović et al. [56]. ALLPATHS-LG takes a unique approach, in that, in addition to long reads, it also incorporates Illumina paired-end reads of two libraries: one with short overlapped fragments, and one with long jumps. Like pure Illumina assembly, short insert read pairs are merged into single “super-reads” for unipath graph generation and the mate pair “jumping” reads are used to fill the gaps in the unipath graph. PacBio reads are subsequently used in order to form consensuses and to patch gaps. While SSPACE-LongRead is mainly a genome scaffolding tool, SPAdes is now capable of hybrid assembly, thanks to a recent update which adds support for taking short and long reads as inputs. Another application of this method on mixed datasets from the yeast genome has been reported by Goodwin et al. [28], who develop a base error correction tool on the ONT reads using Illumina MiSeq reads. The subsequent steps in overlap detection and consensus computation are similar to PBcR.

There are a few assembly pipelines developed or adapted specifically for nanopore sequencing data, as shown in Table 2. Evaluation of assembly methods and tools is difficult as the nanopore sequencing platform is still in the early stages of development and data sources are limited. Sović et al. [56] has presented a comparison report on hybrid and non-hybrid methods for *de novo* assembly from MinION *E*. *coli* reads. Evaluated on five assembly pipelines including LQS, Falcon, PBcR, ALLPATHS-LG, and SPAdes, the assembly quality and continuity varies with read depth and tools used. However, lack of PacBio data for comparison is one of the weak points in the study.

**Figure 4:**
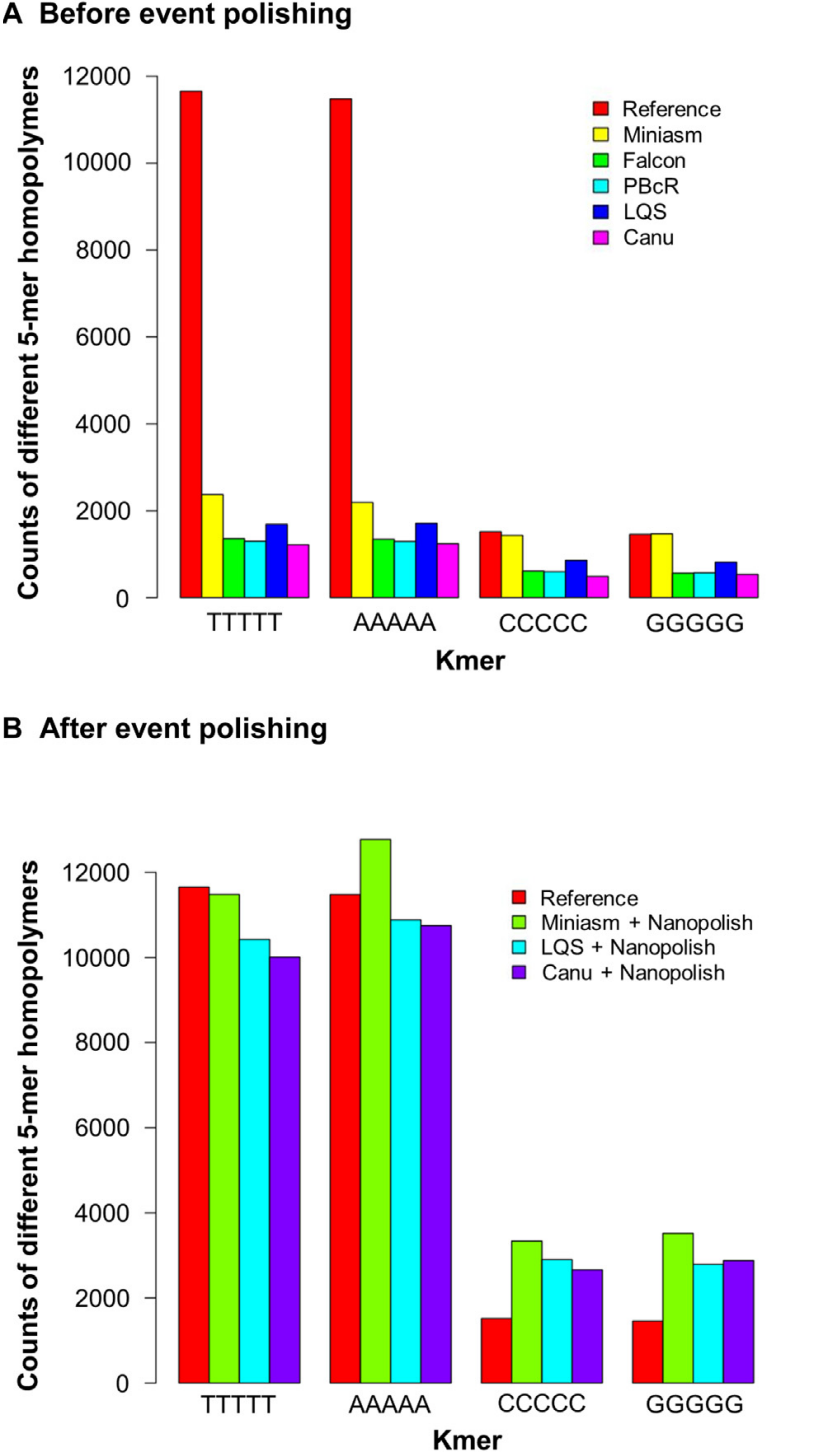
**Counts of 5-mers from the reference and the assembly before and after polishing using event data** **A.** Counts of 5-mer homopolymers before event polishing. **B.** Counts of 5-mer homopolymers after Nanopolish using event data.

To assess the performance of assemblers and the effect of data characteristics on genome assembly, we selected 5 datasets and 6 assemblers for a comparison study. The datasets include both MinION and PacBio reads at different coverages (Table 3). The 20× MinION reads are a subset of 48× reads from [13], while the 20× PacBio reads were selected from a set of 25× *E*. *coli* K12 MG1655 reads downloaded from http://wgs-assembler.sourceforge.net/wiki/index.php/PBcR. The Illumina MiSeq reads were sequenced by Goodwin and her colleagues [28]. To achieve the best assembly, we either tested the parameters ourselves or followed suggestions from other users. A file containing information about how we ran the pipelines is provided as a supplementary file (SF1) and is available at [ftp://ftp.sanger.ac.uk/pub/users/zn1/ont/ont-comparison.pdf]. Mismatches, short indels, and average identity with respect to the reference were estimated using the dnadiff tool from MUMmer [57]. As shown in Table 4, compared with the reference sequence, no global misjoin errors were found in any of the reported assemblies. LQS produced the assemblies with highest consensus base quality before polishing, while the fastest assembler was Miniasm, which omits the process of base error correction on raw sequencing reads. Based on the selected datasets, however, the overall winner is the Canu assembler in terms of contig continuity and computational resources used. Consensus base accuracy in the nanopore assemblies is notably lower than in those using the PacBio reads, likely due to the misrepresentation of homopolymers in the ONT reads. In order to increase the accuracy level, a polishing process using Nanopolish [17] could be run. Figure4**A** shows the under-representation of homopolymers (TTTTT, AAAAA, CCCCC and GGGGG) for the 5 assemblies including Miniasm, Falcon, PBcR, LQS, and Canu, compared to the reference assembly. It is of note that the under-representation problem is significantly improved after Nanopolish has been run (Figure4**B**). This is also shown in the improved Nanopolished assembly accuracies (Table 4).

As shown in Table 4, the hybrid assemblies are only from SPAdes, which uses both long (ONT or PacBio) reads and Illumina short reads. SPAdes assemblies have a higher base accuracy than the nanopore- or PacBio-only assemblers, due to the use of highly accurate Illumina reads. On the other hand, the excellent continuity of the SPAdes assemblies is attributed to the use of the long reads. When running SPAdes on the pure MiSeq data, the assembly is still very accurate (99.99%), but is very fragmented, with 318 contigs and an N50 of 170 kb.

## Other applications

### Diagnostic application

When disease outbreaks occur, it is important to be able to rapidly obtain genome sequences in order to assess the patterns of viral evolution, to investigate transmission chains, or to validate diagnostic assays [58], [59], [60], [61]. It would be difficult to use a Sanger sequencer or find an NGS platform that could match the MinION in terms of its rapid deployability and reliability under field conditions. Additionally, in some places it may not be feasible, perhaps for political or logistical reasons, to export samples to laboratories capable of performing the required analyses. The compact MinION device therefore offers fantastic opportunities to sequence the genome quickly in these less favorable locations and conditions. The device can be used immediately upon arrival in the outbreak area, as it does not require any special setup or calibration procedures. More importantly, the device can be used as a rapid diagnostic tool for management of outbreaks of various diseases due to the rapid data turnaround. Quick et al. [60] reported a landmark diagnostic investigation during the Ebola outbreak in Guinea, west Africa. In order to extract enough DNA for the study, the designed sequencing protocol used a targeted reverse transcription-PCR, and DNA sequences were amplified to cover the Ebola virus (EBOV) genome. A real-time genomic surveillance system was developed for the various tasks of sequencing, which consisted of three MinION devices, four laptop computers, and consumables. In total, they made 148 MinION runs, covering 142 samples. Rapid turnaround is the key for such projects. It was reported that it only took 15–60 min to perform the sequencing process, with the whole workflow completed in less than 24 h, including amplification, library preparation, and MinION run. The study itself and the protocol developed has well demonstrated the potential of nanopore sequencing in areas where sequencing would not otherwise have been possible. Future diagnostic support could revolutionize the ability of the researchers working in the public health sector to perform sequencing during future disease outbreaks. Other diagnostic applications have also been reported, including prediction of antibiotic resistance by sequencing the genomes of *Staphylococcus aureus* and *Mycobacterium tuberculosis* [62]; real-time strain typing and analysis of antibiotic resistance [63]; and aneuploidy detection in prenatal and miscarriage samples with sequencing reads generated in less than 4 h [64] just to name a few. Please visit https://publications.nanoporetech.com for an up-to-date list of applications and publications using ONT sequencing.

### Structural variation

Structural variation (SV), as an important type of genetic variants, includes insertions, deletions, duplications, inversions, and large-scale structural rearrangements. Currently, variants of size ⩾ 50 bp in length are all considered as SVs [65], [66], [67], [68]. Driven by constant advances of sequencing technologies, various analysis methods have been developed to identify SVs. While substitutions and short indels could be detected relatively easily by read alignment, identification of SVs in large size is difficult when using NGS short sequencing reads. Dealing with such situation often needs to employ indirect evidence such as disturbance of read mapping, mapping coverage, and mapping breakpoints. There is rich literature on method development and data collections for SVs [69], [70]. As the NGS market is dominated by the Illumina pair-end sequencing [68], algorithms developed and datasets archived for SVs are mainly for Illumina short reads. With advances in single-molecule sequencing, however, efficient identification of SVs could be substantially enhanced in the genomics community, as long read sequencing platforms such as PacBio and Oxford Nanopore are playing an increasingly important role in genome sequencing.

To demonstrate nanopore sequencing’s ability to detect SVs, Norris et al. [18] designed an experiment to see how well a set of 10 well-characterized SVs could be detected. These SVs included 2 interstitial deletions, 4 translocations, and 4 inversions. SNP microarrays and whole-genome sequencing (WGS) were first used to identify these SVs, which were confirmed by PCR and Sanger sequencing across the junctions afterward. Barcodes were used to generate libraries for all 12 PCR amplicons (10 SV samples + 2 control samples), which were then multiplexed in one MinION flowcell run. In total, this run produced 2.5 megabytes of sequence bases with 3987 2D reads from 194 of 512 channels. The reads were 640-bp long on average, which is about the full length of the PCR products. Although the yield was low and the base error rate was high (86%), all 10 SVs were correctly detected with a wild-type sample that are confirmed to contain no variation serving as control. As the authors noted in the paper, the identification of exact breakpoints is still challenging. Although the SVs detected were all of the correct type, their locations were imprecise, being shifted by a few bases. Improvements are obviously needed, either in the development of bioinformatics tools, or in the reduction of base errors from the sequencing instrument.

## Differences between the PacBio and ONT sequencing platforms

Notably, there are a number of differences between PacBio and ONT platforms even in terms of sequencing methods. While the PacBio platform relies on the method of sequencing-by-synthesis, in which single molecules of DNA polymerase are observed as they synthesize a single molecule of DNA, nanopore-based technologies detect DNA bases by monitoring the transit of a DNA molecule through a hole and measuring variation in electric currents or optical signals. During SMRT sequencing, subreads can be generated, resulting in multiple base coverage on a given base. For nanopore sequencing, however, the base coverage from data stream for each pore is either 1 or 2 (1D or 2D read), as described in detail earlier in this review. One striking feature of nanopore sequencing is the under-representation of common homopolymers in the raw reads. If 5-mers are used for base-calling, the maximum length for homopolymers will be 5 bp, *i*.*e*., there are no 6-mers or longer homo-kmers such as AAAAAA or TTTTTT in the data, since additional bases identical to the ones in the initial 5-mer would not cause a change in the ion current when passing through the pore. Table 5 lists under-represented and over-represented 5-mers for a sequencing dataset of *E. coli* [13]. However, most of the missing homopolymers can be recovered from the event data by Nanopolish [17]. It can be seen from Figure4A that the copy numbers of the 5-mers TTTTT and AAAAA are significantly lower than that of the reference sequences for all the assemblies. After Nanopolish, the copy numbers for these 5-mers are increased, which become close to those seen in the reference (Figure4B). For G- and C-rich 5-mers like GGGGG and CCCCC, it appears that Nanopolish could over-correct them, resulting in more copies of G- or C-rich 5-mers than actual (Figure4B).

We outline below other major differences between ONT and PacBio platforms in terms of instrument size, read accuracy, and read length.

(1) Device size. PacBio platforms are bulky and require a large initial investment. This means that it could only be feasible for them to be owned and operated by large, established sequencing centers. Furthermore, they require a reasonably high level of support throughout the entire sequencing process, again meaning that they are far better suited to sequencing centers, where this kind of support will already be in place. Nonetheless, PacBio platforms have relatively low operating costs when assessed on a per-sample basis. On the other hand, the MinION platform sits in direct contrast, being both far smaller and requiring a lower initial investment than PacBio platforms, making them more accessible to smaller laboratories. Furthermore, the MinION’s applications in real-time sequencing allow for a more general analysis on the samples than that offered by PCR.
(2) Read lengths. The read length from both the PacBio and MinION platforms can be increased by experimental methods, say, for example, by increasing the fragment sizes of the input DNA. Table 3 shows the read lengths of MinION and PacBio data used for our assembly comparison. It can be seen that the mean read length from PacBio is longer than that from MinION. It should be noted that the actual length obtained may vary from run to run, depending on a number of factors, such as DNA samples, quality, and library preparation methods. The longest full length MinION 2D read aligned to reference can be over 45 kb [13]. Nanopore sequencing technology is less likely to have inherent limitations in terms of potential read length, as it is not based on synthesis. When assessed by the proportion of their lengths fully aligned with the reference sequences, reads obtained from the MinION outperform short reads [76], therefore having a higher probability that a sequence can be aligned to a given region of the genome.
(3) GC bias. For ONT, 1D reads from the MinION sequencer have reported base accuracy of 65%−75%, while higher quality 2D reads have base accuracy at 80%−88% [11], [12], [13]. The base error rates for PacBio reads are slightly better, reportedly in the range of 10%−15% [14]. It is common to observe sequencing difficulties in the GC-biased regions, where base accuracy is lower and read coverage is associated with higher variation or less representation, comparing to non-GC-biased regions. Both Ashton et al. [11] and Karlsson et al. [72] observed higher error rates in G/C mononucleotide repeats and therefore further evaluation of the MinION system’s ability to sequence extremely GC-rich regions is needed.

## Discussion and future prospects

The MinION device and the data produced using it have had a number of reported successful applications in disease surveillance, such as the Ebola outbreak in west Africa [60], [61]. For this purpose, the real-time feature is critical in order to swiftly establish progression patterns by monitoring the spread of a disease. The fast output data streamed by the device is a current squiggle, not a DNA sequence string. To speed up data collection for analysis, Loose et al. [71] developed a method which can map the squiggle data directly to a reference sequence in ‘squiggle space’. To achieve a desirable read depth for a given analysis, it would take 49 min to carry out the standard base-calling technique. However, the desired read coverage is obtained in just 15 min using squiggle mapping. This increase in speed would make big differences in medical environments. On another front, David et al. [73] have developed an open source base-caller for offline data processing, which could have profound impact on applications at places where there is a lack of stable electricity supply and reliable internet access.

Library preparation plays an essential role in determining the quality and usability of the data produced by the MinION, and much effort is going into making the process automated and reproducible. A MinION Starter kit includes a library preparation kit, which prepares purified genomic DNA, amplicons, or cDNA ready for loading onto the MinION in less than 2 h. As different applications require different lengths of DNA to be sequenced, users are able to obtain different read length profiles using different library preparation methods. ONT is developing VolTRAX—a small device designed to convert an original biological sample into a form ready for use in a nanopore sequencing device without the need for human intervention. The device can be directly connected to the MinION sequencer. However, quality of the data, usability, and flexibility of VolTRAX remain to be fully tested by the community. For most applications, traditional library preparation kits are routinely selected on extracted DNA.

Current applications of ONT data are mainly on small genomes for variation detection, genome assembly, *etc*. Analysis on large genomes, however, is still limited by the relatively low throughput offered by the MinION device. Although not officially released on the market, ONT has announced a new platform, PromethION [74]. This is a stand-alone benchtop sequencer that is computationally powerful enough to be used for very high throughput real-time analyses. Additionally, PromethION is, like the MinION, able to use the cloud-based analysis service Metrichor. There are 48 flowcells incorporated in PromethION, making it equivalent to 48 MinIONs. Furthermore, there are 3000 nanopores in the nanopore sensor array contained inside each flowcell, giving the instrument 144,000 nanopores in total (281 times the number found in MinIONs). Signal processing is carried out by an application-specific integrated circuit (ASIC) within the device. Multiple samples can be processed separately in different flowcells. It is worth noting that PacBio has also announced a new high-performance platform, Sequel [75], which is expected to launch in late 2016. The total throughput per SMRT cell will initially range 5–10 GB (about 7 times the current throughput), with initial average read lengths of 8–12 kb.

In early March 2016, ONT revealed plans for potential update on MinION technology [76]. The chemistry will change from R7 to R9, by using an *E*. *coli* pore protein CsgG. A new base-caller is being developed and it is moving away from the current HMM-based base-caller to neural network-based base-caller. With these improvements, it aims to achieve a 10% error rate in 1D data and a Q20 for 2D data. In late May 2016, ONT announced SmidgION [77], an iPhone-powered sequencer, which has 256 channels per flowcell and the device is expected to be available in late 2017. The introduction of these high-performance sequencing platforms and devices for automatic library preparations makes it easier to achieve *de novo* assemblies for human-sized genomes at a more affordable price. Moreover, availability of the even smaller device SmidgION will speed up applications in monitoring disease outbreaks and real-time species identification. For the PacBio data, a few *de novo* human assemblies have been reported [46], [47], and computational work is being undertaken to speed up the assembly process. For the ONT data, a significant challenge lying ahead is to develop efficient algorithms, not just for optimizing the use of computational resources for large genomes, but also for obtaining assemblies with high base accuracy using reads with under-represented and over-represented homopolymers.

## Competing interests

ZN is a member of the MinION Access Program (MAP) and has received free-of-charge flowcells and sequencing kits for nanopore sequencing. ZN has had no other financial support from ONT.

## Figures and Tables

**Table 1:** List of analysis tools developed for Oxford Nanopore data

**Name**	**Applications**	**Link**	**Ref.**
BWA	Fast nanopore data tuned alignment tool	https://github.com/lh3/bwa	[19]
GraphMap	Mapper for long and error-prone reads	https://github.com/isovic/graphmap	[25]
LAST	Nanopore tuned alignment tool	http://last.cbrc.jp/	[20]
LINKS	Software tool for long read scaffolding	https://github.com/warrenlr/LINKS/	[26]
marginAlign	Tools to align nanopore reads to a reference	https://github.com/benedictpaten/marginAlign	[27]
minoTour	Real time analysis tools	http://minotour.nottingham.ac.uk/	[24]
nanoCORR	Error-correction tool for nanopore sequence data	https://github.com/jgurtowski/nanocorr	[28]
NanoOK	Software for nanopore data, quality and error profiles	https://documentation.tgac.ac.uk/display/NANOOK/NanoOK	[23]
Nanopolish	Nanopore analysis and genome assembly software	https://github.com/jts/nanopolish	[17]
nanopore	Variant-detection tool for nanopore sequence data	https://github.com/mitenjain/nanopore	[27]
Nanocorrect	Error-correction tool for nanopore sequence data	https://github.com/jts/nanocorrect/	[29]
npReader	Real-time conversion and analysis of nanopore reads	https://github.com/mdcao/npReader	[30]
poRe	Tool for analyzing and visualizing nanopore data	https://sourceforge.net/p/rpore/wiki/Home/	[22]
PoreSeq	Error-correction and variant-calling software	https://github.com/tszalay/poreseq	[31]
Poretools	Nanopore sequence analysis and visualization software	https://github.com/arq5x/poretools	[21]
SSPACE-LongRead	Genome scaffolding tool	http://www.baseclear.com/genomics/bioinformatics/basetools/SSPACE-longread	[32]
SMIS	Genome scaffolding tool	https://sourceforge.net/projects/phusion2/files/smis/	[33]

**Table 2:** List of assemblers for Oxford Nanopore MinION long reads

**Assembler name**	**Algorithms**	**Error correction**	**Link**	**Ref.**
LQS	DALIGNER, Celera OLC	Nanocorrect, Nanopolish	https://github.com/jts/nanopolish	[17]
PBcR	HGAP or BLASR, Celera OLC	PBcR	http://wgs-assembler.sourceforge.net/wiki/index.php/PBcR	–
Canu	MHAP, Celera OLC	Canu	https://github.com/marbl/canu	–
Falcon	String graph, Celera OLC	Falcon	https://github.com/PacificBiosciences/falcon	[48]
Miniasm	OLC	None	https://github.com/lh3/miniasm	[49]
ra-integrate	OLC	None	https://github.com/mariokostelac/ra-integrate/	[56]
ALLPATHS-LG	de Bruijn graph	ALLPATHS-LG	https://www.broadinstitute.org/software/allpaths-lg/blog/?page_id=12	[51]
SPAdes	de Bruijn graph	SPAdes	http://bioinf.spbau.ru/spades	[53]

*Note:* LQS, Loman, Quick and Simpson; PBcR, PacBio Corrected Reads; HGAP, hierarchical genome-assembly process; BLASR, basic local alignment with successive refinement; MHAP, MinHash alignment process; OLC, overlap-layout-consensus.

**Table 3:** Datasets of MinION, PacBio, and MiSeq used for assembly comparison

**Platform**	**Dataset**	**No. of bases**	**No. of reads**	**Mean read length**	**Longest read**	**N50**	**Coverage (×)**	**Identity (%)**
MinION	48× reads	225,086,343	30,364	7413	45,588	8931	48.5	91.41
	20× reads	92,524,457	12,555	7370	45,588	8867	19.9	91.03

PacBio	25× reads	116,263,784	13,124	8859	42,279	14,159	25.0	91.64
	20× reads	90,335,723	10,154	8897	42,279	14,046	19.5	91.54

MiSeq	1263× reads	5,862,970,443	25,758,933	228	302	300	1263	>99.99

*Note:* The reads with lower 20× coverage are subsets of 48× for MinION and 25× for PacBio platforms, respectively. Matching identities for the three sets of data were estimated using the reference assembly.

**Table 4:** Assembly assessment using different tools for three types of reads at different levels of coverage

**Data source**	**Assembly tool**	**No. of assembled bases**	**No. of contigs**	**N50**	**No. of mismatches**	**No. of indels**	**Identity (%)**	**CPU time (h)**
48× MinION reads	LQS	4,636,840	2	4,624,206	1677	41,646	99.06	8662
	Falcon	4,562,630	1	4,562,630	2606	102,872	97.70	16.2
	Canu	4,574,127	1	4,574,127	760	73,623	98.39	20.2
	PBcR	4,567,749	1	4,567,749	982	80,325	98.22	46.4
	Miniasm	4,544,438	1	4,544,438	173,356	330,040	88.90	0.05
	SPAdes	4,638,974	1	4,638,974	255	31	99.99	19.1

20× MinION reads	Falcon	4,137,874	23	2,693,898	4608	107,781	97.29	1.6
	Canu	4,546,300	1	4,546,300	2141	94,233	97.90	6.8
	PBcR	4,398,520	35	1,021,865	1824	88,269	97.95	10.0
	Miniasm	4,526,673	17	2,921,034	173,127	326,625	88.89	0.01
	SPAdes	4,639,128	3	3,108,521	254	31	99.99	24

48× MinION reads with Nanopolish	LQS	4,685,134	2	4,672,162	903	18,464	99.58	375
	Miniasm	4,666,535	1	4,666,535	16,153	51,954	98.54	2540
	Canu	4,654,817	1	4,654,817	1155	18,730	99.57	434

25× PacBio reads	Falcon	4,621,993	2	4,196,046	457	10,898	99.75	1.4
	Canu	4,663,990	1	4,663,990	20	3903	99.91	2.8
	PBcR	4,638,751	2	3,746,511	48	3368	99.93	7.2
	Miniasm	4,830,837	1	4,830,837	81,535	432,590	89.33	0.02
	SPAdes	4,638,975	1	4,638,975	254	30	99.99	19

20× PacBio reads	Falcon	4,036,562	47	145,816	459	12,422	99.68	0.8
	Canu	4,637,297	1	4,637,297	90	7736	99.83	2.6
	PBcR	4,456,708	32	239,659	114	6260	99.86	5.8
	Miniasm	4,777,699	9	917,934	75,726	419,198	89.46	0.01
	SPAdes	4,638,975	1	4,638,975	254	30	99.99	18.9

*Note:* The SPAdes assemblies were obtained using both long and short reads, while other assemblies used only one type of long reads, either MinION or PacBio. Take the dataset 48× MinION as an example, the SPAdes assembly was obtained using 48× MinION reads as well as MiSeq reads, while the assembly of Falcon was generated from MinION reads only.

**Table 5:** Under-represented and over-represented 5-mers of the *E*. *coli* data

**Group**	**Template reads**				**Complement reads**				**2D reads**			
	**Kmer**	**Occurrence/100 bp**		**Diff**	**Kmer**	**Occurrence/100 bp**		**Diff**	**Kmer**	**Occurrence/100 bp**		**Diff**
		**in Ref**	**in Templ**			**in Ref**	**in Compl**			**in Ref**	**in 2D**	
Under-represented	AAAAA	0.247	0.086	−0.161	CGCCA	0.288	0.092	−0.196	TTTTT	0.251	0.047	−0.204
	TTTTT	0.251	0.093	−0.158	AAAAA	0.247	0.055	−0.192	AAAAA	0.247	0.058	−0.189
	CGCTG	0.258	0.104	−0.155	TTTTT	0.251	0.065	−0.186	CAAAA	0.170	0.111	−0.058
	GCTGG	0.279	0.148	−0.132	CACCA	0.184	0.054	−0.130	AAAAT	0.195	0.138	−0.057
	CGCCA	0.288	0.168	−0.120	CCAGC	0.288	0.162	−0.126	AAAAG	0.132	0.081	−0.051
	CCAGC	0.288	0.180	−0.108	CGCTG	0.258	0.135	−0.123	CGCCA	0.288	0.239	−0.049
	GCCAG	0.280	0.173	−0.107	GCCAG	0.280	0.157	−0.122	TAAAA	0.145	0.097	−0.048
	CTGGC	0.278	0.178	−0.100	CAGCA	0.262	0.140	−0.122	TGGTG	0.185	0.138	−0.048
	CAGCA	0.262	0.168	−0.095	CTGGC	0.278	0.159	−0.119	CGCTG	0.258	0.213	−0.046
	CGGCA	0.222	0.129	−0.093	TGGCG	0.275	0.163	−0.112	GCCAG	0.280	0.238	−0.042

Over-represented	ACCCC	0.040	0.136	0.096	ACCCC	0.040	0.143	0.103	CAAAT	0.105	0.164	0.059
	CCCCG	0.055	0.149	0.093	CCCCG	0.055	0.134	0.079	GGGGT	0.039	0.074	0.035
	CCCCC	0.033	0.122	0.089	CCCCA	0.064	0.128	0.065	CCCAA	0.047	0.080	0.033
	CCCCA	0.064	0.138	0.075	CCTAG	0.003	0.066	0.063	TGAAT	0.121	0.154	0.033
	CCTAG	0.003	0.075	0.072	CTGAG	0.050	0.112	0.063	GAAGG	0.094	0.127	0.033
	GCCCC	0.062	0.131	0.069	TACCC	0.073	0.136	0.062	CGGGG	0.054	0.087	0.032
	CTCCC	0.039	0.107	0.067	CCTAA	0.026	0.087	0.061	ACCGT	0.123	0.155	0.032
	TCTAC	0.048	0.113	0.065	GACCC	0.040	0.100	0.060	CGTGA	0.102	0.134	0.032
	TCCCC	0.056	0.121	0.065	TCCCC	0.056	0.115	0.059	GAAGC	0.124	0.156	0.032
	TACCC	0.073	0.138	0.064	TCCTA	0.013	0.071	0.058	AGGCA	0.093	0.124	0.031

*Note:* Poretools was used to extract FASTA sequences for template, complement, and 2D reads from the FAST5 files. The 5-mer counts of the reads of the *E*. *coli* data [13] and of the reference assembly were calculated separately using the oligonucleotide frequency function of the R package Biostring. Frequencies of each 5-mer occurrence in reads per 100 bp were calculated and differences in reads relative to Ref are indicated. Ref, reference assembly; Templ, template read; Compl, complement read.

## Supplementary material

Supplementary material SF1 Detailed information on the datasets and assemblers used in this article.
