# Supplementary material for: Oxford Nanopore MinION Sequencing and Genome Assembly
Source: Genomics Proteomics Bioinformatics. 2016 Sep 17;14(5):265–79. doi: 10.1016/j.gpb.2016.05.004 (PMC5093776; doi:10.1016/j.gpb.2016.05.004)
Supplement: Supplementary material SF1 — Detailed information on the datasets and assemblers used in this article. [file mmc1.docx]

**SF1 Data Analysis**

**Datasets**

We used the following datasets.

*Oxford Nanopore E. coli data*

The Oxford Nanopore (referred as Nanopore hereafter) data used in the analysis are the MARC sample ERA434488, and can be downloaded from: ftp://dcc_marc@ftp.sra.ebi.ac.uk/data/ERA434/ERA434488/oxfordnanopore_native/UCSC_MARC_Phase_Ia_Run_2.tar.gz. The 2D reads have been extracted from the FAST5 files in the ‘pass’ directory using Poretools (<https://github.com/arq5x/poretools>), and they have 48× coverage. In order to test the assemblers, we created a subsample with 20× coverage using randomly selected reads.

*PacBio E. coli data*

We compared the results of the various assemblers when using Nanopore or PacBio datasets. For this task, we used the PacBio FASTA file ecoli_p6_25x.filtered.fasta from [http://wgs­assembler.sourceforge.net/wiki/index.php/PBcR](http://wgs-assembler.sourceforge.net/wiki/index.php/PBcR). We run the assemblers on the entire dataset, which has about 25× coverage. For a better comparison with the Nanopore low coverage dataset (20×) we also run the assemblers on a PacBio subsample with 20× coverage.

*MMiSeq E. coli data*

For the hybrid assembler SPAdes, we used a 1263 × set of MiSeq paired reads, available at [h ttp://schatzlab.cshl.edu/data/nanocorr/](http://schatzlab.cshl.edu/data/nanocorr/).

**A Assemblers**

We tested five pure­Nanopore assembler pipelines.

*LQS*

LQS (<https://github.com/jts/nanopore­paper­analysis>) is a Nanopore­only­pipeline and it uses information provided in the original FAST5 files. We used the settings as described in the related paper [19] and as defined in the makefile provided by the authors: [https://github.com/jts/nanopore­paper­analysis](https://github.com/jts/nanopore-paper-analysis), except for the choice of a more recent Nanopolish commit [82]. Running for more than 8600 CPU hours on the 48× Nanopore set, this was the slowest of the pipelines we tried. Due to its lack of speed, and because the LQS authors themselves deprecated Nanocorrect, the LQS error correction step [(http://simpsonlab.github.io),](file:///C:\Users\zn1\AppData\Local\Microsoft\Windows\Temporary%20Internet%20Files\Content.IE5\JHDCU5K1\(http:\simpsonlab.github.io),) we only ran it for one case: the complete Nanopore dataset (48×). Unless it is explicitly stated, the LQS assembly pipeline does not include the Nanopolish step for a fairer comparison with assemblers without a final polishing stage.

*PBcR*

PBcR (h[:ttps://wgs­assembler.sourceforge.net/wiki/index.php/PBcR](http://wgs-assembler.sourceforge.net/wiki/index.php/PBcR)) is a Celera-based assembler, and was run with the standard Nanopore settings for the 48× Nanopore dataset. For the 25× PacBio dataset, PBcR was run with the standard PacBio settings. For both the Nanopore and the PacBio 20× samples, we chose the low coverage settings suggested in [http://wgs­assembler.sourceforge.net/wiki/index.php/PBcR#Low_Coverage_Assembly](http://wgs-assembler.sourceforge.net/wiki/index.php/PBcR#Low_Coverage_Assembly), as we saw significant improvement in the assemblies when running with these settings.

*Canu*

The Canu (<http://canu.readthedocs.org/>) assembler is able to run without specifying any settings except for the genome expected size, as it can autodetect the available resources and scale the jobs accordingly. For this reason, Canu is the easiest assembler to run out of the ones we tried for this analysis, and furthermore it ranks as one of the best in terms of speed and assembly quality. Similarly to PBcR, Canu is a fork of the Celera Assembler. For the smaller datasets (20× Nanopore and 20× PacBio) we used the ‘low­coverage’ settings suggested on the package’s webpage.

*Miniasm*

Miniasm (<https://github.com/lh3/miniasm>) is a very fast assembler (< 5 min for our *E. coli* datasets) for long and error­prone reads, but it does not perform an initial read error correction so the generated draft assembly has an average identity with the reference genome similar to that of the raw reads. We ran it with the standard settings as suggested by the author.

*Falcon*

Falcon (<https://github.com/PacificBiosciences/FALCON>) is an assembler specifically built for the long and error­prone reads from PacBio platform. As it does not access the raw PacBio h5 files, it can also work with Nanopore reads. We ran it using the makefile provided in the example [hhttps://github.com/PacificBiosciences/FALCON/wiki/Setup%3A­Complete­example](https://github.com/PacificBiosciences/FALCON/wiki/Setup%3A-Complete-example), but we changed the assembly settings to match the ones optimized for Nanopore reads in [61].

We also ran the hybrid assembler SPAdes (<http://bioinf.spbau.ru/spades>) with standard settings on the 1263× MiSeq paired reads and either the long reads from the Nanopore or the PacBio platforms.

Even in the presence of an initial read error correction step, the maximum average identity we observed from pure­Nanopore assemblies was 99.0% with the tested data. In an attempt to increase the accuracy, we ran Nanopolish on a few assemblies from the 48× Nanopore dataset: the LQS assembly (which includes Nanopolish naturally as a step of the pipeline); the assembly from Canu, which provided a good single contig with the second highest average identity after LQS; and the Miniasm assembly, which does not include an error correction step at all. We ran Nanopolish with its standard settings in all these cases. After using Nanopolish, assemblies using LQS and Canu had similar average identities of about 99.6%, while Miniasm’s assembly reached 98.5% average identity. The result for Miniasm is remarkable because the assembly was initially constructed without an error correction step. Unfortunately, as the original Miniasm assembly’s average identity was only around 89%, the Nanopolish step was very slow (taking more than 2500 CPU hours), making the combination Miniasm and Nanopolish highly impractical.
